# The Association between Psychosocial and Age-Related Factors with Adherence to Immunosuppressive Therapies after Renal Transplantation

**DOI:** 10.3390/jcm11092386

**Published:** 2022-04-24

**Authors:** Justyna Zachciał, Izabella Uchmanowicz, Michał Czapla, Magdalena Krajewska, Mirosław Banasik

**Affiliations:** 1 Department of Nephrology and Transplantation Medicine, Wroclaw Medical University, 50-556 Wroclaw, Poland; justyna.zachcial@umw.edu.pl (J.Z.); magdalena.krajewska@umw.edu.pl (M.K.); miroslaw.banasik@umw.edu.pl (M.B.); 2 Department of Nursing and Obstetrics, Faculty of Health Sciences, Wroclaw Medical University, 51-618 Wroclaw, Poland; izabella.uchmanowicz@umw.edu.pl; 3 Laboratory for Experimental Medicine and Innovative Technologies, Department of Emergency Medical Service, Wroclaw Medical University, 51-616 Wroclaw, Poland; 4 Faculty of Nursing, Group in Research in Care (GRUPAC), University of La Rioja, 26006 Logroño, Spain

**Keywords:** kidney transplantation, medication adherence, immunosuppressants, acceptance of illness, quality of life

## Abstract

Renal transplantation (RT) is the optimal renal replacement treatment approach in terms of patient survival and high quality of life. Proper adherence to medication is essential in order to prolong graft life and patient survival. This study aimed to investigate the effects of psychosocial factors and age-related declines on adherence in kidney transplant recipients. Methods: This was a cross-sectional study of kidney transplant recipients, based on regression analysis. Patient adherence was assessed with the Basel Assessment of Adherence with Immunosuppressive Medication Scale (BAASIS). Psychosocial and age-related variables were measured with the World Health Organization’s quality of life questionnaire (WHOQoL-BREF), the Mini-Mental State Examination (MMSE), the Hospital Anxiety and Depression Scale (HADS), the Acceptance of Illness Scale (AIS), and the Tilburg Frailty Indicator (TFI). Results: A simple linear regression model indicated that the significant predictors of self-reported adherence (*p* < 0.05) were age, time since transplant, and anxiety and cognitive functions. For problems with implementing immunosuppressive medication, logistic regression models showed that gender, age, retirement status, hypercholesterolemia, and cognitive impairment were the most significant predictors (*p* < 0.05). However, after controlling for other predictors in the multiple regression models, anxiety and cognitive ability no longer predicted treatment adherence to immunosuppressive medication. Conclusions: Renal transplantation is the most effective therapy in chronic renal failure patients. Proper adherence to immunosuppressive therapy is critical to prolonging graft and person survival. Our study shows that occupational status more significantly influences adherence to the implementation of treatment in kidney transplant recipients.

## 1. Introduction

Renal transplantation (RT) is currently the most cost-effective treatment for end-stage renal failure [1]. It involves a lower medical cost, achieves a higher patient survival rate [2], and offers a better health-related quality of life (HRQoL) [3] if compared with dialysis. Several studies show that the HRQoL of patients who underwent a functioning renal graft is higher than patients receiving hemodialysis treatment. Although the HRQoL advantages of RT are well established [4], it is also noticeable that life after renal transplant presents negative aspects as well, such as the strict immunosuppressive regimen and its side effects, frequent medical visits, rejection episodes, infections, and the uncertainty and anxiety concerning the potential loss of the graft [5].

All of these listed factors influence medical adherence, which reflects the degree to which a patient follows directions regarding treatments and medications prescribed. It is a significant predictor of outcome, particularly among those requiring chronic therapies. Nonadherence results in disease progression, increased healthcare costs, and even premature death [6,7]. In transplantation, the association of nonadherence with late rejection and graft loss makes it a critical determinant of patient outcome [7,8]. A recent meta-analysis reported that non-adhering patients had a sevenfold higher risk of graft failure than adhering patients [9]. When considering adherence specifically following transplant, Laederach-Hofmann and Bunzel [10] found that one-fifth of all transplanted patients do not take their medication as prescribed. Taken together, these findings demonstrate the need to study the specific factors connected with nonadherence following kidney transplantation in order to encourage adherence.

Nonadherence largely depends on sociodemographic and medical factors, including the patient’s age, education and income levels, the requirements of the treatment, the side effects of immunosuppressants, and the time since transplantation [10,11]. Some reports clearly show a significant relationship between immunosuppressive medication adherence and the age of transplant patients. For instance, Greenstein and Seagull [12] found that elders are more adherent to immunosuppressive therapy than younger patients. Similar results were found by Weng et al. [13] in their research. Greenstein and Seagul also found that medication adherence becomes less likely as more time passes after a renal transplant [12].

One critical aspect of adherence allows medical personnel, caregivers, and researchers to see and explore it more in-depth. Adherence to a medication regimen is typically defined as the degree to which patients take the medication their healthcare providers prescribe for them. At the same time, the word “adherence,” which is preferred over “compliance,” suggests that the patient is proactively following and adjusting to the treatment plan according to an agreement between the patient and physician [14], wherein the patient’s input is crucial to the success of the treatment. This relation indicates a wide range of psychological factors that may also influence adherence [15].

Achille and colleagues found that perceived stress and psychological distress contributed to nonadherence. In contrast, a feeling of being indebted to the donor led renal transplantation patients to follow their medication regimen [16]. The literature also lists other psychological factors involved in patients’ nonadherence, such as anxiety, depression, transplant-related stress, and a health locus of control [17,18,19]. Greater adherence, on the other hand, was associated with better social functioning and positive feelings towards the doctors [20].

The main aim of the study was to investigate the protective factors and related risk of adherence in a Polish population of transplant recipients. The present study investigated the hypothesized associations of the psychosocial and age-related factors with adherence, based on a cross-sectional design.

## 2. Materials and Methods

### 2.1. Study Design

The study was conducted on a sample of 190 patients, including 89 women and 101 men, with a mean age of 61.65 years (SD = 12.11). Patients recruited for the research study were being treated at Wroclaw Medical University Hospital. Recruited patients were admitted to the Nephrology Department between 2018 and 2020. Participation in the survey required patients that be 18 years old or older, have a diagnosis of end-stage chronic kidney disease, and be the recipient of a kidney transplant.

The present study was approved by the Bioethical Committee of the Wroclaw Medical University (KB-789/2018). Participation in the research was voluntary and the data were anonymized. Prior to taking part in the study, the participants were familiarized with the main objectives of the study. After being educated about the study, the respondents provided their informed consent to participate. After that, all the consenting individuals were asked to fill in the questionnaires during hospitalization, in the presence of a nurse.

The questionnaire was delivered to 190 consecutive patients in our out-patient clinic.

### 2.2. Instruments

#### 2.2.1. Tilburg Frailty Indicator (TFI)

The TFI scale measures frailty syndrome. It consists of fifteen questions, divided into three domains: (1) the physical domain (8 questions) addresses physical health, unexplained weight loss, difficulty walking, balance, hearing problems, vision problems, strength in hands, and physical fatigue (range 0–8 points); (2) the psychological domain (4 items) addresses cognition, depressive symptoms, anxiety, and coping (0–4 points); (3) the social domain (3 questions) addresses living alone and social relationships (range 0–3 points). Eleven of the TFI items are “yes” or “no” questions, while four items use three-point response options (yes, no, and sometimes). Each item is scored from 0 to 1, so the total score for frailty ranges from 0 to 15 points. Higher scores indicate a higher degree of frailty [21].

#### 2.2.2. Hospital Anxiety and Depression Scale (HADS)

The HADS is a 14-item questionnaire used to assess the presence and severity of anxiety and depression [22]. The questionnaire consists of seven questions about anxiety and seven questions about depression, which are scored on a scale of 0 to 3. It takes 2–5 min to complete the HADS. Cut-off points are available for screening purposes; for example, a score of 8 or more for anxiety has a specificity of 0.78 and a sensitivity of 0.9. For depression, it has a specificity of 0.79 and a sensitivity of 0.83. The HADS deals with non-physical symptoms, and can therefore be helpful in diagnosing depression in patients with poor physical health [23]. The HADS scores were interpreted according to the HADS manual, whereby a score of 0 to 7 indicates no symptoms, a score of 8 to 10 indicates a borderline condition, and a score of 11 to 21 indicates the presence of disorder [24].

#### 2.2.3. Acceptance of Illness Scale (AIS)

The Acceptance of Illness Scale (AIS) assesses the degree of disease acceptance. The AIS was developed by Felton et al. [24] and adapted to the Polish population by Juczyński [25]. This measurement evaluates the patient’s adaptation to their health state. It examines a patient’s acceptance of illness without experiencing the negative emotions and reactions associated with the disease. The questionnaire consists of eight statements relating to the negative consequences of poor health, such as limitations imposed by the illness, dependence on others, low self-esteem, and a lack of self-efficacy. The items are rated by a 5-point Likert scale from 1 (“strongly agree”) to 5 (“strongly disagree”), with a maximum of 8 and a minimum of 40 points. Low scores indicate less ability to adapt to and accept one’s illness, accompanied by psychological discomfort; high scores indicate acceptance of one’s illness and limited negative emotions connected with the disease. The AIS has been validated by many clinical studies on cancer, rheumatoid arthritis, diabetes, hypertension, and other chronic illnesses. The tool is considered reliable (α = 0.83 before treatment and 0.81 after treatment). The test-retest reliability coefficient was found to be 0.69 [26].

#### 2.2.4. The WHOQOL-BREF Quality of Life Scale

WHOQOL-BREF is a 26-item, cross-cultural, self-report scale used to quantify the health-related quality of life of patients. The WHOQOL-BREF consists of 24 items that cover four domains of quality of life: (1) psychological (6 items); (2) physical health (7 items); (3) social relationships (3 items); (4) environmental (8 items). In addition, the scale includes one item that refers to the overall quality of life, and one item that covers general health. All items are rated using a 5-point scale, whereby a higher score indicates a better quality of life [26].

#### 2.2.5. The Mini-Mental State Examination (MMSE)

The MMSE scale provides a brief, quantitative and objective evaluation of various aspects of cognitive function, regarding orientation to time and place, immediate recall, short-term verbal memory, calculation, language, and construct ability. The measure yields a total score of 30. The assessment of the depth of dementia is based on the sum of the points, whereby 27–30—normal score, 24–26—cognitive impairment without dementia, 19–23—mild dementia, 11–18—moderate dementia, 0–10—severe dementia [27].

#### 2.2.6. Basel Assessment of Adherence with Immunosuppressive Medication Scale (BAASIS)

The BAASIS scale is commonly used to assess adherence to immunosuppressive drugs in transplant recipients [28]. It verifies whether the patient is taking the medication as prescribed [1]. BAASIS records adherence over the last 4 weeks and consists of five questions on implementation and persistence of adherence: missed dose, medication interruptions (skipping two or more doses), time deviation (>±2 h), and dose change or discontinuation of medication without consulting the physician. Non-compliance is defined as a “yes” answer to any questions. In addition to the general interpretation of the BAASIS scale, which allows for the categorization of subjects as adherent or nonadherent, responses were divided into subscales: (1) problems in implementation (their presence is indicated by at least one “yes” answer in questions 1A, 1B, 2 and 3); (2) the discontinuation of immunosuppressive drugs (answer “yes” in question 5); (3) self-reported adherence on a scale of 0% (never took the medication as prescribed) to 100% (always took the medication as prescribed).

### 2.3. Adherence Educational Approach

Our transplant center consists of two departments: surgical and internal. The first surgical is Department of General, Vascular, and Transplantation Surgery, and the internal Recipients are transferred for surgical procedure after preparation clinical but also education by transplant nephrologist. Transplant nephrologists take care of the patients also before transplantation. On the second day after transplantation the recipients are transported to the internal department which takes over care again till discharge day. During this time transplant nephrologists together with dedicated nurses educate the patients. Additionally, recipients receive a folder with a book and other materials which explain why adequate taking immunosuppressants are essential for not only graft survival but also patients’ survival. Such education performed by transplant nephrologists together with a dedicated nurse in our opinion is the crucial way for adequate education. During this education nephrologists explain not only the danger connected with not taking immunosuppressants but also skipping the dose, taking itat another time, or with unsuitable foods that have a bad influence on the immunosuppressant’s level in the blood. The time of education is adjusted for each transplant recipient and is about 2–4 h of transplant nephrologists’ care and about one–two hours of the dedicated nurse’s during first stay before discharge from hospital. During the first three months after transplantation, the patient is in the out-patient clinic usually 3–5 times during which not only the immunosuppressant level and routine blood tests are performed but also the knowledge of adherence is verified and reminded. The immunosuppressant’s level is very important to confirm the adequate dose which also is connected with adherence. An inappropriate level of immunosuppressant is a reason for reeducation and also an explanation of the consequences such as graft loss and return to dialysis.

### 2.4. Statistical Analysis

Quantitative analysis was carried out by computing the mean (M), standard deviation (SD), median, quartiles, minimum, and maximum. The analysis of qualitative variables provided frequencies in absolute numbers and percentages. Simple linear and multiple regression analyses were then performed. The normality of the distribution of the regression’s residuals was checked with the Shapiro–Wilk statistics. A higher VIF indicates a more problematic f collinearity between predictors. The 95% confidence intervals (CI) were estimated for the regression coefficients and the quality of the linear models was assessed with coefficients of determination (R2). Multicollinearity was checked using the VIF indicator, and was found not to exist (all VIFs were below 5). In addition, the homogeneity of the residuals was assessed with the Breush–Pagan test.

In the analysis, statistical significance was set at a *p*-value of 0.05. The statistical analysis was done in R Statistical Package, version 4.0.3.

## 3. Results

Table 1 consists of sociodemographic and clinical data. Table 2 consists of a statistical description of frailty (TFI), acceptance of illness (AIS) quality of life (WHOQoL BREF), depression and anxiety (HADS), and cognitive impairment (MMSE), and adherence (BAASIS) scores for the study group. Table 3 consists of an interpretation of the raw scores for frailty (TFI), quality of life (WHOQoL BREF), depression and anxiety (HADS), cognitive impairment (MMSE), and self-assessed adherence (BAASIS) in the study group.

### 3.1. Predictors of Adherence to Immunosuppressive Medications (BAASIS)

#### 3.1.1. Predictors of Self-Reported Adherence to Immunosuppressive Medications

A series of simple regression analyses were performed to examine how sociodemographic, clinical, and psychological variables predicted self-reported adherence to immunosuppressive medications. The simple linear regression showed that the significant predictors of self-reported adherence (*p* < 0.05) were age, time since transplant, and anxiety and cognitive functions (see Table 4).

In particular, age correlated positively with the self-reported adherence variable (b = 0.13; *p* < 0.001), indicating that older patients self-reported being more compliant with recommendations. There was a negative association between time since transplant and self-reported adherence (b = −0.13; *p* = 0.02). Each year passing since the transplant reduced self-rated adherence by an average of 0.131 percentage points on the BAASIS scale. The regression parameter for HADS anxiety was b = −0.25 (*p* = 0.029). Each score on this subscale decreased self-rated treatment adherence by an average of 0.25 percentage points. The regression analysis showed that better cognitive functioning, as indicated by the MMSE, lowered self-assessed treatment adherence by 0.35 percentage points on average; b = −0.351; *p* = 0.015. There were also significant associations between selected sociodemographic and clinical variables and self-reported adherence. Patients in rural areas were more adherent to recommendations (b = 2.019; *p* = 0.028) than people from urban areas. Respondents in rural areas rated their adherence to recommendations higher (b = 2.019; *p* = 0.028) than people from urban areas. Patients who were retired (b = 5.993; *p* < 0.001), annuitant (b = 5.027; *p* < 0.001), and unemployed (b = 6.667; *p* = 0.038) reported better compliance than working participants. It was also found that patients with comorbid hypertension rated their adherence higher (b = 2.888; *p* = 0.006) than patients without hypertension.

Multiple regression was performed to examine the predictors of self-reported adherence to immunosuppressive medications, given the variables selected in the simple regression analyses (see Table 4). Retired and pensioner status, as well as comorbid hypertension, were significant predictors of self-reported adherence. These obtained coefficients indicated that retirees scored 4.11% higher than active subjects (b = 4.11, *p* = 0.007), and annuitants scored on average 3.51% higher than employed patients (b = 3.51, *p* = 0.012). The regression analysis indicated that patients with comorbid hypertension scored higher on the adherence self-assessment scale, by an average of 2.74%, than those with no hypertension (b = 2.74, *p* = 0.013).

#### 3.1.2. Predictors of Implementation of Medical Adherence to Immunosuppressive Medications (BAASIS)

The logistic regression models show that the factors of gender, age, retirement status, hypercholesterolemia, and cognitive impairment (MMSE) were the most significant predictors (*p* < 0.05) of problems with implementing immunosuppressive medication (see Table 5). The analysis indicates that males were 2.33 times more likely to have problems implementing medical recommendations than females (OR = 2.333; 95%CI: 1.073–5.07). Age was associated with a decreased likelihood of problems with implementing recommendations (OR = 0.967; 95%CI: 0.941–0.994). Each year of life led to less nonadherent behaviors, suggesting that younger respondents were more prone to have difficulties with adherence. Retired individuals were less likely to have issues with adherence implementation than working respondents (OR = 0.137; 95%CI: 0.053–0.355). Professionally active individuals were 7.30 times more likely to have problems following recommendations than retirees. Hypercholesterolemia co-morbidity also increased the likelihood of having problems following the recommendations 3.138-fold compared to non-sufferers (OR = 3.138, 95%CI: 1.122–8.774). Surprisingly, the regression analysis indicated that each point on the MMSE increased the likelihood of nonadherence by about 1.17-fold (OR = 1.173; 95%CI: 1.015–1.357). The outcomes suggest that better cognitive functioning likely leads to issues with implementing recommendations.

Multiple logistic regression was conducted to examine the independent predictors and adherence implementation issues, while keeping constant all the other predictors in the regression model. This analysis allowed us to estimate the effects of each individual variable via other variables in the model (see Table 5). The regression analysis (*p* < 0.05) indicated that the factors of retirement status and hypercholesterolemia were significant independent predictors for problems with immunosuppressive medication implementation. Retired individuals were less likely to have issues with recommendations than employed individuals (OR = 0.087; 95%CI: 0.015–0.51). Employed individuals were 11.5 times more likely than retired individuals to implement adherence behaviors. Patients with comorbid hypercholesterolemia were 5.774 times more likely to have issues with implementation than non-sufferers (OR = 5.774; 95%CI: 1.242–26.847).

## 4. Discussion

Adherence to immunosuppressive therapy and medical recommendations is crucial for short- and long-term outcomes in transplant recipients. In this study, nearly every fifth patient-reported problems with adherence (18.95%), and 1.58% stopped taking their medication. Another study showed that the rate of self-reported nonadherence 6 weeks after RT was about 17%, but this value increased up to 27% 6 months after transplantation [29]. These results suggest that patients’ perceptions of medical adherence implementation may change over time since the transplant procedure. It is worth mentioning that the mean time since transplantation in our study was 9.58 years (SD = 7.42).

Several studies have suggested that a long time after transplant is associated with more frequent adherence failures [12,30,31,32,33]. This may reflect that transplant recipients become less adherent to recommendations over time. Some researchers explained this via the less attentive patient supervision of the healthcare system [34]. Therefore, some studies suggest that decreased adherence may be the effect of a lack of continuous reinforcement, regarding the importance of compliance, over the long term. Vasquez et al. noticed that the longer the post-transplant period, the better patients feel, and the less they perceive themselves as being sick [32]. One study suggests that participants get used to taking medications over time and eventually no longer regard it as problematic [35].

The time elapsed seems to be essential for predictors of nonadherence. Different predictors of nonadherence may arise depending on the time since transplantation. A meta-analysis of reasons for nonadherence after kidney transplantations [36] indicated that potentially modifiable factors, compared to unmodifiable ones, are more influential in the development of nonadherence to medication. This conclusion is not consistent with the results of our study. Self-reported adherence was influenced by rather unmodifiable factors, such as age, occupational activity, and comorbidities. Specifically, problems with adherence to immunosuppressive medications were associated with such predictors as sex, age, occupational activity, and the comorbidity of hypercholesterolemia. This could be caused by the relatively long time that has elapsed since transplantation in our study. We suppose that some psychological factors, such as anxiety, depression, quality of life, and acceptance of the illness, are essential to the adherence to immunosuppressive treatment recommendations shortly after transplantation. Due to the psychological and physical changes in the requirements of early and late post-transplant patients, some other psychological predictors of adherence may be found for patients several years after transplantation.

The most significant portion of the effort of early post-transplant patients is exerted on post-surgery recovery, rehabilitation, and adjustment to lifestyle changes and new treatment habits. Therefore, certain factors are more relevant immediately after transplantation, while others grow in importance over time [31]. For example, a change in how long the graft is expected to last was a significant predictor of nonadherence 6 months after transplantation [31]. Such beliefs, in turn, may not be significant in the case of patients who have already lived for many years with a transplanted kidney. In turn, it was shown that among late post-transplant recipients, less control over treatment and more symptom distress were associated with nonadherence [37]. Thus, it is interesting to investigate how patients’ needs, perceptions, and emotions related to illness and treatment transform over time. The factors that influence treatment adherence seem to be crucial to planning appropriate interventions and improving compliance in post-transplant patients. Primarily, future research should focus on identifying adherence-related factors that are vulnerable to alteration via psychological or medical interventions in early and late post-transplant recipients.

In our study, of the psychological factors, only anxiety was a significant predictor of self-reported treatment adherence, and cognitive functioning was significant for both self-reported treatment adherence and problems with the implementation of treatment recommendations. However, after controlling for the effects of other variables in the regression models, anxiety and cognitive ability no longer predicted treatment adherence. It is possible that the models included variables that moderate the effects of anxiety and cognitive functioning on adherence to treatment recommendations. In other words, other variables could affect the strength of the relationships between independent variables (anxiety and cognitive functioning) and the dependent variable (adherence). For example, the relationship between anxiety and adherence might be different in younger vs. older patients. The obtained associations of cognitive functioning and self-reported adherence with problems with the implementation of treatment recommendations were not intuitive. Namely, better cognitive functioning was associated with worse patient adherence and more problems with the implementation of medical recommendations. Moreover, in our study, age was related to better adherence and fewer problems with implementing recommendations. Our findings are not consistent with the results of other studies. For example, Gelb et al. [34] found that better cognitive functioning, as measured by everyday problem-solving tests, was predictive of higher self-reported medication adherence. It was also shown that older post-transplant patients showed greater nonadherence to medication than younger recipients [34]. Some researchers suggest that general cognitive and physical declines related to older age impact nonadherence [36]. Thus, the relationships between age, cognitive functioning, and adherence are still unclear.

Further research is necessary to establish which cognitive domains improve adherence, and which make it worse. Moreover, the impact of cognitive functioning may be moderated by other variables, such as social support. For example, patients with cognitive impairments supported by relatives who monitor their medication implementation may be more adherent. On the other hand, cognitive dysfunctions may interfere with treatment adherence in patients who are not supported, and their adherence thus depends entirely on these factors.

The multiple regression analysis showed that retirees and annuitant patients reported higher compliance than employed patients. In fact, employed patients may be exposed to a busy lifestyle, which disturbs the regular medication routine [31,38,39]. In our study, retired patients had fewer problems with implementing the recommendations than the employed recipients. On the other hand, some studies have shown that unemployment is more common in nonadherent patients [40]. However, other possible factors explaining the inconsistent results include the financial inaccessibility of immunosuppressive medications, and the healthcare politics of the country [41,42].

We do believe that education is the crucial way to the adequate taking of prescribed immunosuppressants. Transplantation is thought of mainly as a typical surgical procedure and most surgical centers take care of patients in the early period after transplantation till a first discharge but very often also over a longer time. Surgeons have firstly not enough time because they should be in the theatre and secondly, they focus on the technical part of the surgical procedure which is without doubt the most important at the beginning. We decided therefore to organize the procedure in another way described in Section 2.3 (adherence to educational approach) with the dominant educational role of transplant nephrologists and dedicated nurses.

Younger recipients required more time and explanation; therefore, we tried to check their immunosuppressant level and also asked for check-ups more often when we suspected the possibility of nonadherence. A very busy person is usually better organized and aware of the lack of time and the consequences but if they were in potential trouble, we also tried to perform additional appointments and reeducate them. In our country, patient do not pay for additional out-patient clinic appointments so we may individualize visits. Patients who are retired or annuitants may have more time but on the other hand, they usually have memory problems. We tried to keep contact with their family or ask for participation in visits to improve adherence. We do believe that individualization is an appropriate approach.

Problems with implementing the recommendations of immunosuppressive medications were more likely to be present in patients with a comorbidity of hypercholesterolemia. Additionally, patients with hypertension attained higher scores on the self-assessment adherence scale than those without hypertension. Somatic co-morbidities have been neglected in the literature [39]. However, some studies using comorbidity parameters have indicated that patients with somatic comorbidities reported lower levels of medication adherence [41,43]. Our findings indicate that some comorbid illnesses might affect adherence, while others may not. Future studies should explore which comorbid somatic diseases interfere with treatment adherence in post-transplant patients.

Our study has some limitations. We employed a self-report scale, which includes socially desirable answers. Due to this fact, this tool may lead to an overestimation of the prevalence of adherence in post-transplant patients [44]. On the other hand, self-report measures are considered economical and easy to use [29]. Some studies have indicated that self-reported nonadherence is significantly correlated with nonadherence measured by electronic monitoring—the gold standard procedure [33]. Therefore, a self-report scale is considered a valuable measure of adherence [33,44].

## 5. Conclusions

Renal transplantation is the most effective therapy in chronic renal failure patients. This treatment approach yields the highest individual survival and quality of life rates. However, very strict medication requirements must be met after the transplant. Proper adherence to immunosuppressive therapy is critical to prolonging graft and patient survival.

Our study shows that occupational status more significantly influences adherence to the implementation of treatment in kidney transplant recipients. Each patient should be analyzed individually in terms of both drug regimen optimization and patient-related factors, as mentioned above. These concerted efforts on the part of multi-disciplinary teams may well prevent nonadherence incidents and provide transplant patients with the best care and a long life.

## Figures and Tables

**Table 1:** Basic clinical and sociodemographic data of study respondents (*n* = 190).

Feature		Mean (SD)	Median (Q1–Q3)
Age (years)		61.65 (12.11)	64 (59–70)
Time since transplantation (years)		9.58 (7.42)	8 (2.25–16)
		***n***	**%**
Sex	Female	89	46.84%
	Male	101	53.16%
Place of residence	Urban area	135	71.05%
	Rural area	55	28.95%
Marital status	Alone	38	20.00%
	In a relationship	152	80.00%
Education	Primary	16	8.42%
	Vocational	47	24.74%
	Secondary	79	41.58%
	Higher	48	25.26%
Occupational activity	Employed	36	18.95%
	Retired	101	53.16%
	Annuitant	50	26.32%
	Unemployed	3	1.58%
Comorbidities ^a^			
	Diabetes	45	23.68%
	Hypercholesterolemia	18	9.47%
	Hypertension	153	80.53%
	Rheumatic diseases	27	14.21%
	Others	33	17.37%
tacrolimus concentration < 5 (ng/mL) or cyclosporine concentration < 100 (ng/mL)	Never	72	37.89%
	Rarely	50	26.32%
	Sometimes	25	13.16%
	Often	20	10.53%
	Very often	23	12.11%

Note: ^a^—the answers to multiple-choice questions, thus the overall percentages exceed 100.

**Table 2:** Statistical description of frailty (TFI), acceptance of illness (AIS) quality of life (WHOQoL BREF), depression and anxiety (HADS), cognitive impairment (MMSE) and adherence (BAASIS) scores in the study group (*n* = 190).

Scale	Mean	SD	Median	Min	Max	Q1	Q3
AIS	30.87	8.03	32	8	40	25	38
HADS							
Anxiety	5.02	3.64	4	0	17	2	7.75
Depression	3.41	3.29	3	0	15	1	5
WHOQoL BREF							
Overall QOL	3.78	0.74	4	1	5	3	4
General health	3.23	0.99	3	1	5	3	4
Physical Health	13.94	2.46	14	7	20	13	15
Psychological Health	15.51	2.19	15.5	9	20	14	17
Social Relationships	15.9	2.56	16	8	20	15	17
Environment	15.43	1.94	16	10	20	14	17
TFI							
Overall	5.3	2.74	5	0	15	3	7
Physical components	2.99	2.19	3	0	8	1	5
Psychological components	1.45	0.96	2	0	4	1	2
Social components	0.86	0.66	1	0	3	0	1
MMSE	26.86	2.89	28	17	30	25	29.75
BAASIS: self-assessed adherence	97.95	5.75	100	60	100	100	100

Abbreviations: AIS—Acceptance of Illness scale; HADS: Anxiety, HADS: Depression—Hospital Anxiety and Depression Scale; WHOQoL BREF—quality of life questionnaire; QOL—quality of life; TFI—Tilburg Frailty Indicator; MMSE—the Mini-Mental State Examination; BAASIS—Based Assessment of Adherence with Immunosuppressive Medication Scale.

**Table 3:** Interpretation of raw scores for frailty (TFI), quality of life (WHOQoL BREF), depression and anxiety (HADS), cognitive impairment (MMSE) and self-assessed adherence (BAASIS) in the study group (*n* = 190).

Scale	Scores	Interpretation	*n*	%
TFI	0–4	No frailty	80	42.11%
	≥5	Frailty	110	57.89%
WHOQoL BREF	NA.			
HADS: Depression	0–7	Normal	164	86.32%
	8–10	Borderline abnormal (borderline case)	20	10.53%
	11–21	Abnormal (case)	6	3.16%
HADS: Anxiety	0–7	Normal	142	74.74%
	8–10	Borderline abnormal (borderline case)	33	17.37%
	11–21	Abnormal (case)	15	7.89%
MMSE	0–10	Severe dementia	0	0.00%
	11–18	Moderate dementia	1	0.53%
	19–23	Mild dementia	23	12.11%
	24–26	Cognitive impairment without dementia	57	30.00%
	27–30	Normal score	109	57.37%

Abbreviations: AIS—Acceptance of Illness scale; HADS:Anxiety, HADS: Depression—Hospital Anxiety and Depression Scale; WHOQoL BREF—quality of life questionnaire; QOL—quality of life; TFI—Tilburg Frailty Indicator; MMSE—the Mini-Mental State Examination; BAASIS—Based Assessment of Adherence with Immunosuppressive Medication Scale.

**Table 4:** The simple and multiple regression analyses of relationships between sociodemographic, clinical, and psychological variables and self-assessment adherence.

Variable		Simple Linear Regressions				Multiple Regression			
		b	95%CI		*p*	b	95%CI		*p*
Sex									
Female		ref.				ref.			
Male		−0.67	−2.31	0.97	0.424	−0.579	−2.252	1.095	0.499
Age (years)		0.13	0.065	0.196	<0.001 *	0.048	−0.057	0.153	0.37
Place of residence									
Urban area		ref.				ref.			
Rural area		2.019	0.235	3.803	0.028 *	1.849	−0.166	3.864	0.074
Marital status									
Alone		ref.				ref.			
In a relationship		−1.151	−3.194	0.891	0.271	−0.783	−2.932	1.365	0.476
Education									
Primary		ref.				ref.			
Vocational		−0.247	−3.482	2.988	0.881	0.786	−2.461	4.033	0.636
Secondary		−1.54	−4.603	1.524	0.326	0.287	−2.783	3.358	0.855
Higher		−2.875	−6.101	0.351	0.082	0.49	−3.032	4.012	0.785
Occupational activity									
Employed		ref.				ref.			
Retired		5.993	3.969	8.018	<0.001 *	4.106	1.159	7.052	0.007 *
Annuitant		5.027	2.747	7.307	<0.001 *	3.506	0.809	6.203	0.012 *
Unemployed		6.667	0.399	12.935	0.038 *	5.798	−0.767	12.363	0.085
Time since transplantation (years)		−0.131	−0.24	−0.022	0.02 *	−0.087	−0.213	0.038	0.176
Comorbidities									
Diabetes	No	ref.				ref.			
	Yes	1.408	−0.509	3.326	0.152	0.424	−1.555	2.403	0.675
Hypercholesterolemia	No	ref.				ref.			
	Yes	−1.108	−3.902	1.687	0.438	−0.706	−3.524	2.112	0.624
Hypertension	No	ref.				ref.			
	Yes	2.888	0.86	4.917	0.006 *	2.743	0.592	4.894	0.013 *
Rheumatic diseases	No	ref.				ref.			
	Yes	−0.5	−2.846	1.847	0.677	−0.255	−2.842	2.333	0.847
Others	No	ref.				ref.			
	Yes	0.687	−1.474	2.849	0.534	0.707	−1.422	2.836	0.516
Tacrolimus concentration < 5 (ng/mL) or cyclosporine concentration < 100 (ng/mL)									
Never		ref.				ref.			
Rarely		−1.435	−3.515	0.645	0.178	1.143	−1.045	3.331	0.308
Sometimes		−1.675	−4.297	0.947	0.212	−1.06	−3.813	1.692	0.451
Often		−1.325	−4.18	1.53	0.364	−0.285	−3.213	2.642	0.849
Very often		−1.571	−4.276	1.135	0.257	−0.112	−2.923	2.699	0.938
AIS		0.002	−0.1	0.104	0.968	0.025	−0.111	0.161	0.72
HADS									
Anxiety		−0.25	−0.473	−0.027	0.029 *	−0.302	−0.625	0.022	0.07
Depression		−0.028	−0.277	0.222	0.827	0.092	−0.31	0.494	0.655
WHOQoL BREF									
Overall QOL		−0.736	−1.849	0.377	0.197	−0.937	−2.178	0.303	0.141
General health		−0.564	−1.394	0.265	0.184	−0.735	−1.712	0.241	0.142
Physical health		−0.087	−0.421	0.247	0.61	0.141	−0.407	0.689	0.615
Psychological health		0.008	−0.368	0.384	0.967	0.03	−0.612	0.672	0.928
Social relationships		0.006	−0.314	0.327	0.969	0.176	−0.239	0.59	0.407
Environment		−0.082	−0.505	0.341	0.703	−0.05	−0.63	0.529	0.865
TFI									
Overall		0.177	−0.122	0.476	0.247	−0.022	−0.388	0.344	0.906
Physical components		0.32	−0.053	0.693	0.095				
Psychological components		−0.397	−1.249	0.455	0.362				
Social components		0.38	−0.858	1.617	0.548				
MMSE		−0.351	−0.632	−0.071	0.015 *	−0.184	−0.503	0.136	0.262

Abbreviations: b—linear regression unstandardized parameter; AIS—acceptance of illness; CI—Confidence Interval, HADS, Anxiety, HADS Depression—Hospital Anxiety and Depression Scale; WHOQoL BREF—quality of life questionnaire; QOL—quality of life; TFI—Tilburg Frailty Indicator; MMSE—the Mini-Mental State Examination. *—*p* < 0.05.

**Table 5:** The simple and multiple logistic regression analysis of sociodemographic, clinical, and psychological variables for problems with the implementation of treatment recommendations.

Variable		Simple Logistic Regressions				Multiple Logistic Regression			
		OR	95%CI		*p*	OR	95%CI		*p*
Sex									
Female		ref.				ref.			
Male		2.333	1.073	5.07	0.032 *	2.337	0.805	6.783	0.119
Age (years)		0.967	0.941	0.994	0.017 *	1.006	0.949	1.068	0.831
Place of residence									
Urban area		1	ref.			1	ref.		
Rural area		0.429	0.167	1.097	0.077	0.239	0.056	1.016	0.053
Marital status									
Alone		1	ref.			1	ref.		
In a relationship		1.311	0.503	3.422	0.579	0.528	0.13	2.15	0.373
Education									
Primary		1	ref.			1	ref.		
Vocational		1.786	0.193	16.549	0.61	0.941	0.065	13.648	0.965
Secondary		4.113	0.507	33.39	0.186	1.843	0.15	22.701	0.633
Higher		5.571	0.667	46.508	0.113	2.145	0.136	33.782	0.587
Occupational activity									
Employed		1	ref.			1	ref.		
Retired		0.137	0.053	0.355	<0.001 *	0.087	0.015	0.51	0.007 *
Annuitant		0.395	0.154	1.013	0.053	0.422	0.093	1.919	0.264
Unemployed		0.7	0.058	8.445	0.779	1.095	0.05	23.886	0.954
Time since transplantation (years)		1.024	0.976	1.075	0.329	1.028	0.954	1.107	0.468
Comorbidities									
Diabetes	No	1	ref.			1	ref.		
	Yes	0.46	0.167	1.263	0.132	0.606	0.148	2.486	0.486
Hypercholesterolemia	No	1	ref.			1	ref.		
	Yes	3.138	1.122	8.774	0.029 *	5.774	1.242	26.847	0.025 *
Hypertension	No	1	ref.			1	ref.		
	Yes	0.812	0.336	1.965	0.644	0.761	0.208	2.788	0.68
Rheumatic diseases	No	1	ref.			1	ref.		
	Yes	0.968	0.34	2.757	0.951	0.883	0.183	4.248	0.876
Others	No	1	ref.			1	ref.		
	Yes	1.188	0.47	3.002	0.715	2.205	0.635	7.659	0.213
Tacrolimus concentration < 5 (ng/mL) or cyclosporine concentration < 100 (ng/mL)									
Never		1	ref.			1	ref.		
Rarely		1.098	0.424	2.841	0.848	0.32	0.075	1.364	0.124
Sometimes		2.353	0.828	6.685	0.108	3.223	0.67	15.509	0.144
Often		1.25	0.355	4.402	0.728	1.513	0.28	8.175	0.631
Very often		0.75	0.192	2.93	0.679	0.458	0.078	2.695	0.388
AIS		0.985	0.943	1.03	0.514	0.968	0.892	1.05	0.431
HADS									
Anxiety		1.035	0.938	1.141	0.494	1.035	0.846	1.268	0.735
Depression		1.048	0.942	1.166	0.386	1.203	0.94	1.541	0.143
WHOQoL BREF									
Overall QOL		1.629	0.928	2.859	0.089	2.063	0.884	4.812	0.094
General health		1.229	0.84	1.799	0.288	1.552	0.857	2.808	0.147
Physical Health		1.037	0.893	1.202	0.636	1	0.705	1.419	1
Psychological Health		1.027	0.869	1.213	0.759	1.144	0.777	1.683	0.496
Social Relationships		1.076	0.93	1.245	0.326	1.144	0.89	1.469	0.294
Environment		1.004	0.833	1.211	0.965	0.877	0.597	1.289	0.505
TFI									
Overall		0.983	0.859	1.124	0.797	1.096	0.881	1.362	0.411
Physical components		0.946	0.798	1.12	0.518				
Psychological components		1.196	0.812	1.762	0.365				
Social components		0.932	0.536	1.623	0.805				
MMSE		1.173	1.015	1.357	0.031 *	1.038	0.857	1.257	0.7

Abbreviations: OR—odds ratio; ref.—referral level of variable; AIS—acceptance of illness; CI- Confidence Interval; HADS, Anxiety, HADS, Depression—Hospital Anxiety and Depression Scale; WHOQoL BREF—quality of life questionnaire; QOL—quality of life; TFI—Tilburg Frailty Indicator; MMSE—the Mini-Mental State Examination. *—*p* < 0.05.

## Data Availability

The data can be accessed by contacting the corresponding author.
